# Observations upon the Importance and Value of a Knowledge of the Collateral Branches of Medicine and Surgery in Connection with Dentistry

**Published:** 1847-10

**Authors:** James Robinson

**Affiliations:** London.


### ARTICLE IV.

*Observations upon the Importance and Value of a Knowledge of the Collateral Branches of Medicine and Surgery in connection with Dentistry.* By JAMES ROBINSON, D. D. S., London.

[Continued from vol. vii, p. 324.]

HAVING pursued the subject thus far, and demonstrated the advantages that the dental practitioner may derive from an acquaintance with anatomy and its intimate relations, physiology and pathology—having shown that a knowledge of medicine and surgery (in which these sciences are included) is essential to *his* reputation and the safety of his patient, I shall proceed to prove that the other collateral branches of medicine are of much, if not of equal importance—that the only legitimate way of studying the art *is as a whole*, and that the chain by which all these necessary branches of knowledge are connected, must be rendered insufficient by the absence of any, even the most unimportant of its links.

Dental surgery may be considered as an offset of medicine, modified it is true, and requiring other and important additional qualifications; but still it is so interwoven and mixed up with that science that no one can practice the one, at least scientifically and successfully, who has not a liberal acquaintance with the other, for who can comprehend the superstructure, who is ignorant of the foundation, or reason on effects, if unacquainted with the causes from which they arise? or the different processes by which they may have been modified?

Possessed of this knowledge, the dentist is enabled to give breadth to his views—to assimilate the local to the general treatment, and take advantage of every resource afforded either by nature or art—while on the contrary, without it, his practice must be uncertain and empyrical. He will have no real data to go upon, and if successful, it will be the result of chance rather than the legitimate effect of scientific reasoning.

It is a curious fact that with some wise heads a profession ranks in dignity, in an inverse ratio, to its mental regularity, thus the *pure* surgeons consider every thing but the use of the scalpel or amputating knife (in the employment of which, a butcher would in all human probability be infinitely more expert), as beneath their dignity, and a knowledge of the collateral branches of medical science as supererogatory if not absolutely disgraceful. In the same way, the science of dental surgery has become to be considered, at least by some *intellectual* individuals, as an art *per se*, or as merely an *operative* one, while the fact directly the reverse is the case, and the scientific practitioner, who is armed at all points, will not only possess superior discrimination in the diagnosis of diseases, but infinitely more resources in combatting them, will be able in many cases to overcome difficulties, ameliorate suffering, and *avoid* operations that would be inevitable in the hands of one less liberally educated.

While medicine, properly so called, teaches the value of the different symptoms and appearances of the various forms of disease, and enables us to distinguish the one from the other, materia medica, which simply means the *materials* of *medicine*, enables us, as far as that is concerned, to supply remedial measures. That these sciences are intimately blended and connected with each other, is so self-evident, that materia medica must be considered rather as a subordinate, though important branch of medicine, than as a distinct and separate science.

Of what use would it be for a physician gravely to tell his patient that his pain proceeded from gravel or inflammation, unless prepared with remedies to combat it, or *vice versa*, what would he gain, by an intimate acquaintance with the properties and doses of every drug contained in the materia medica, or all the chemical remedies in the pharmacopoeia to boot, unless his knowledge of medicine taught him in what cases they might be employed with advantage.

Thus, then, while anatomy teaches the different parts of the human body, and physiology their duties—while pathology points out their diseased actions, and organic changes, a combination by which we are able to discriminate sympathetic and symptomatic affections from structural diseases, materia medica, chemistry and botany form the three links in the chain of remedies, and it is of vital importance to the dental practitioner that these links should be perfect.

I do not mean for one moment to assert, that a man may not practice dental surgery; nay, that he may not gain a reputation, or, at all events, a fortune, without these acquirements; unfortunately, the converse of this is of too common occurrence, for the public are, generally speaking, more willing to patronise specious and showy pretension, rather than scientific desert—and the consequence is, that unless a practitioner have some more high and honorable feeling than the mere love of gain, or an avidity for meretricious praise; unless he be determined to sacrifice ease, comfort and self-interest to his profession, and be content to rank rather as a scientific practitioner than a fashionable dentist, with all the pecuniary emolument thereunto belonging, he may be tempted to neglect all but the common and vulgar necessities of his profession, and seeing others *get on* with a small amount of knowledge, fancy that he may do the same, and that a liberal and general knowledge of medicine, is, to say the least, unnecessary.

That a liberal acquaintance with materia medica, is essential to the scientific dentist is indisputable. If he have only a contracted knowledge of the subject, he may, it is true, order an application to an unsound tooth, or prescribe a lotion for the mouth, but even putting out of the question what may be called general treatment—he will not, he cannot be able to select the best or most *appropriate* means, or employ them in a scientific or artistic manner; he may do as his *teacher* has instructed him, but he will not be able to practice with any reliance on his own judgment. Should he venture out of the beaten track, it will be with fear and trembling to himself, and uncertainty, if not danger to his patient. Like a friend of ours, who always added sweet spirits of nitre to a febrifuge mixture, because his master had done so before him. If an acquaintance with materia medica is essential to the dental practitioner, an acquaintance with chemistry is still more so; for as the former, generally speaking, refers to vegetable, or, as they are called simple remedies, the latter involves those of a more complicated character, having, independently of these medical effects, peculiar and specific actions on each other, and, although, in many cases these actions are evidenced by visible and palpable changes, in others they are so obscure, that unless a practitioner be perfectly acquainted with them, he may, while prescribing what he imagines a judicious course, be merely giving what would be called, in vulgar parlance, chip in porridge.

There are scarcely two, of what may be strictly called chemical medicines, that can be mixed without some change in one or both: Thus, magnesia which is, in its simple state, an absorbent earth, has its action changed and reversed by the action of fire; while the addition of sulphuric acid forms the compound called Epsom salts, and yet, *calcined* magnesia may be mixed with the acid, and the new compound formed, without any visible intimation of the changes. Again, preparations of quicksilver mixed with alkalies, undergo a change of color, and it is from this cause that the amalgams used for stopping the teeth, under the names of anodyne cement, &c. &c., so frequently blacken and discolor them.

Chemical remedies, generally speaking, have a more decided and concentrated action than those of a vegetable kind, which renders them at the same time more effective if used with discretion, but at the same time more dangerous in the hands of ignorance and empiricism; for while they are more certain in their action, they are more liable to have that action interfered with and changed by admixture.

Thus, then, in the treatment of disease, whether dental or general, or a combination of both, it is necessary not only to ascertain what the disease is, but what remedies will counteract it, but to select those best adapted to the purpose; for though, under certain circumstances we may be obliged to employ one remedy, we are bound, when we have the choice, to select the most appropriate, and this can only be done by a perfect acquaintance with materia medica and chemistry.

Take a case of poisoning, which, though unconnected, will illustrate my position. A person has taken sulphuric acid, magnesia will neutralise and convert it into a purgative salt. If the carbonate of magnesia be employed, a considerable quantity of gas will be evolved in the stomach, producing distension and flatulence; while calcined magnesia will neutralise the acid without this inconvenience. Still, in a case of necessity, we should use the one in preference to waiting for the other. These cases, however, do not occur in dental practice in which there is always time to select the remedy best adapted to the purpose.

